# Dr. R. S. Myers

**Published:** 1904-11

**Authors:** 


DR. R. S. MYERS, of Clarence Center, N. Y., died at his residence, September 30, 1904. Dr. Myers was a graduate of the University of Vermont, class of 1875.

